# The Rational Use of Disinfectants

**Published:** 1907-02-09

**Authors:** Thomas Divine

**Affiliations:** Huddersfield


*To the Editor of* The Hospital.

### THE RATIONAL USE OF DISINFECTANTS.

*To the Editor of* The Hospital.

Sir,—I notice in your issue of February 2 that it is claimed by another firm that Jeyes' Company were not the pioneers of the method of standardising disinfectants by bacteriological examination. The appreciation shown by this firm for the bacteriological method is indicative of its value, and they therefore claim that it has been for over fifteen years a foremost principle with them. That they derived the principle from Jeyes' method of standardising is indisputable, since for five years previously to their entertaining the subject Jeyes had distributed a large literature dealing with this aspect of the question, dating from the time when Professor Attfield, in 1887, conducted a careful series of experiments on the relation of Jeyes' Fluid to micro-organisms. He pronounced the Fluid to be a true germicide, a true disinfectant, and a true antiseptic, and stated that no microbe would long resist its action. Each succeeding year found new investigators, and Koch, Esmarch, Eisenberg (in 1888), Max Kortün, and a host of others made valuable experiments with Jeyes' Fluid, known on the Continent as Creolin, although now denominated Cyllin. This was five years prior to the discovery of Izal; therefore Izal must have followed Jeyes' Company in giving a guarantee "in definite and scientific terms."

The *British Medical Journal* in 1888 had reported that, "compared with carbolic acid, it was found that a 3 per cent. mixture of Creolin (*i.e.* Cyllin) killed the spores of the anthrax bacillus in a period of time in which a carbolic mixture of nearly three times the strength had no effect at all." The same might be said of the cholera bacillus and of all the other micro-organisms known at that time. The standardisation of disinfectants was originally introduced and developed by Jeyes' Company, and the modern methods now adopted are merely a development of those of 1887.

Your correspondents contend that the "pure culture" method of standardisation is quite useless in standardising disinfectants which will be used in impure media. This is as it may be; it all depends upon the disinfectant; but they have not yet appreciated the fact that Messrs. Jeyes have demonstrated that it is easy to determine the action of Cyllin in any form of organic material: for example, urine, pus, or sputum. If pus is to be disinfected, the disinfectant is standardised by a measure of albumen; where sputum is concerned the proportion can be established by a standard of mucin. The Rideal-Walker method of testing the activity of disinfectants in the presence of organic matter was originally suggested by Jeyes' Company. In such tests they show the actual results obtained when different organic diluents are used in place of water, and find that whereas other disinfectants show a considerable drop in efficiency, Cyllin does not vary more than 1.5.

Your correspondents allege that the Hebling and Passmore pamphlet "upheld the chemical as against the bacteriological method." This is stating but half a fact. Here are some of their exact words: "Without possessing the property of activity against bacteria to a very high degree, the disinfectant is no disinfectant. Its action must be prompt and sure." "The bacteriological method has undoubtedly furnished very important and valuable results, but is of little use unless supported by practical experience." "Chemical methods are more reliable if the germicidal power of the various chemicals contained in the disinfectants are once known." I should think there is nothing in these statements Messrs. Newton, Chambers and Co. would find any scruple in subscribing to even at this date.

For my own part I think the Rideal-Walker method of standardising disinfectants will stand a good deal more criticism than it has yet been subjected to. I see no objection to the substitution of milk instead of distilled water, as recently suggested by Mr. Winter-Blyth, provided that a standardised milk be employed.

A committee of experts are at present considering the best means of standardising disinfectants, and also what the standard should be. A report by this committee will probably soon be issued, but until then public bodies, in my opinion, would be well advised (in the interest both of efficiency and economy) to be guided in their purchases of disinfectants by the findings of the Rideal-Walker method of standardisation.—Yours faithfully,

Thomas Divine, M.D., D.P.H., F.C.S.

Huddersfield, February 4, 1907.

---

The return issued on Saturday last giving the statistics of pauperism during the last quarter in 1906, shows a rather lower figure than in 1905; the figures supplied by the Local Government Board being 22.7 in 1906 and 23.3 in 1905 per 1,000 of population. The proportion of people receiving poor relief is now 23.1 per 1,000, as against 23.6 per 1,000 in the previous year. In London there has been a slight diminution in the number of persons relieved throughout the quarter, a result due to the decrease of outdoor paupers.

